# State of equity: childhood immunization in the World Health Organization African Region

**DOI:** 10.11604/pamj.supp.2017.27.3.12114

**Published:** 2017-06-21

**Authors:** Rebecca Mary Casey, Lee McCalla Hampton, Blanche-philomene Melanga Anya, Marta Gacic-Dobo, Mamadou Saliou Diallo, Aaron Stuart Wallace

**Affiliations:** 1 Epidemic Intelligence Service, Centers for Disease Control and Prevention, Atlanta, USA; 2 Global Immunization Division, Centers for Disease Control and Prevention, Atlanta, U.S.A; 3 World Health Organization Regional Office for Africa, Brazzaville, Republic of Congo; 4 Department of Immunization, Vaccines and Biologicals, World Health Organization, Geneva, Switzerland; 5 Division of Data, Research and Policy, United Nations Children’s Fund, New York, USA

**Keywords:** Vaccination, immunization coverage, World Health Organization African region

## Abstract

**Introduction:**

In 2010, the Global Vaccine Action Plan called on all countries to reach and sustain 90% national coverage and 80% coverage in all districts for the third dose of diphtheria-tetanus-pertussis vaccine (DTP3) by 2015 and for all vaccines in national immunization schedules by 2020. The aims of this study are to analyze recent trends in national vaccination coverage in the World Health Organization African Region andto assess how these trends differ by country income category.

**Methods:**

We compared national vaccination coverage estimates for DTP3 and the first dose of measles-containing vaccine (MCV) obtained from the World Health Organization (WHO)/United Nations Children’s Fund (UNICEF) joint estimates of national immunization coverage for all African Region countries. Using United Nations (UN) population estimates of surviving infants and country income category for the corresponding year, we calculated population-weighted average vaccination coverage by country income category (i.e., low, lower middle, and upper middle-income) for the years 2000, 2005, 2010 and 2015.

**Results:**

DTP3 coverage in the African Region increased from 52% in 2000 to 76% in 2015,and MCV1 coverage increased from 53% to 74% during the same period, but with considerable differences among countries. Thirty-six African Region countries were low income in 2000 with an average DTP3 coverage of 50% while 26 were low income in 2015 with an average coverage of 80%. Five countries were lower middle-income in 2000 with an average DTP3 coverage of 84% while 12 were lower middle-income in 2015 with an average coverage of 69%. Five countries were upper middle-income in 2000 with an average DTP3 coverage of 73% and eight were upper middle-income in 2015 with an average coverage of 76%.

**Conclusion:**

Disparities in vaccination coverage by country persist in the African Region, with countries that were lower middle-income having the lowest coverage on average in 2015. Monitoring and addressing these disparities is essential for meeting global immunization targets.

## Introduction

In 2012, the World Health Assembly endorsed the Global Vaccine Action Plan 2011-2020 (GVAP), which calls on all countries to reach and sustain 90% national coverage and 80% coverage in all districts for the third dose of diphtheria-tetanus-pertussis (DTP3) containing vaccine by 2015 and 90% national coverage and 80% coverage in all districts for all vaccines included in national immunization schedules by 2020 [1]. The GVAP also calls for a focus on equity in vaccination coverage through reducing the gap in coverage between low-income countries and high-income countries, and by reducing pockets of low sub-national vaccination coverage. Within the WHO African Region (AFR), a majority of countries have historically been classified by the World Bank as low-income [2], and immunization services in many countries usually operate within a relatively resource-constrained environment compared to high-income countries. Most of the 47 African Region countries are the focus of heavy investments from foreign donors, including Gavi, the Vaccine Alliance, which has disbursed at least US$6 billion during 2000-2016 for vaccine introductions and health system strengthening activities in 37African Region countries [3, 4].

No published analyses have described recent trends in vaccination coverage, equity and vaccine introductions among African Region countries in light of the substantial investments made in these countries over the past 16 years. Examining how these trends differ by country income status and by Gavi-eligibility status may provide useful insight into gaps to be addressed in future work by the countries and their external partners. Additionally, as global partners such as Gavi, WHO and UNICEF look to further support countries in reaching GVAP goals, identifying countries which have outperformed their peers may also yield useful lessons learned for strengthening immunization systems within resource-constrained settings.

The aims of this study are to analyze recent trends in national and subnational vaccination coverage in the African Region, to assess how these trends differ by country income category and Gavi-eligibility, to review the coverage achieved with recently introduced vaccines in African Region countries and to identify low-income African Region countries which outperform their peers in reaching and sustaining high levels of vaccination coverage.

## Methods

### Data sources

Global, regional and national vaccination coverage estimates were obtained from the WHO/UNICEF estimates of national immunization coverage (WUENIC) released in July 2016 [5, 6]. These data provide coverage estimates for multiple routine vaccinations, including third dose of DTP-containing vaccine (DTP3), third dose of *Haemophilus influenza* type b vaccine (Hib3), third dose of polio vaccine (Pol3), measles-containing vaccine first dose (MCV1) and second dose (MCV2), third dose of pneumococcal conjugate vaccine (PCV3), first dose of rubella-containing vaccine (RCV1), and last dose of rotavirus vaccine (Rota-last) during 1980-2015. Country-reported estimates of the proportion of districts that have reached 80% vaccination coverage in a given year were obtained from annual country-reported data collected through the WHO/UNICEF Joint Reporting Form on Immunization (JRF) process and made available for public use [7]. Information on vaccine introductions was obtained from data made publicly available by WHO [8].

National estimates for the number of surviving infants were obtained from the United Nations Population Division (UNPD), 2015 revision [9]. National estimates of gross national income per capita (GNI per capita) and categorization of countries by national income level from 1990 to 2015 were obtained from the World Bank [10]. Information on whether a country was eligible forGavi funding was obtained from sections of the Gavi website on countries’ eligibility for and transitions from Gavi support [11, 12].

### Definitions

Country income categories were defined using World Bank historical criteria for low-income, lower middle-income, upper middle-income, and high-income countries within the given year of analysis [2]. The World Bank uses GNI per capita, calculated using the World Bank Atlas method, to categorize countries by income level. For instance, in 2015, low-income countries were defined as those with a GNI per capita of $1,025 or less, lower middle-income countries were those with a GNI per capita between $1,026 and $4,035, upper middle-income countries were those with a GNI per capita between $4,036 and $12,475, and high-income countries were those with a GNI per capita of $12,476 or more.

Gavi-eligible countries are defined as those that were eligible (GNI per capita ≤US$ 1,000)for Gavi funding in Phase 1, commencing in 2000. This group includes 37 countries in AFR. Despite changes in the criteria for countries’ eligibility for Gavi support, 35 countries in the African Region were still eligible for Gavi support in 2015 [11].

Vaccination coverage is defined as the proportion of surviving infants in a given year that received the vaccine. DTP1–3 dropout is defined as the proportion of infants who received the first dose of DTP vaccine (DTP1) but did not receive the third dose of DTP vaccine (DTP3). According to WHO Immunization in Practice guidelines, a dropout rate of 10% or higher indicates that challenges may exist with ensuring that children who start a country’s recommended schedule are able to receive all doses of recommended vaccination series [13,14].

### Data analysis

For several vaccines and numbers of doses (DTP3, Pol3, MCV1, MCV2), we evaluated trends in African Region coverage compared to global-level coverage between 2000 and 2015. We compared national vaccination coverage estimates, the proportion of districts in a country reported to reach ≥90% DTP3 coverage, and the number of unvaccinated children for DTP3 across African Region countries for 2015, as well as the trend in subnational coverage from 2010 to 2015. We calculated the number of unvaccinated children in each country by using the UNPD estimates for surviving infants in 2015 and the WUENIC 2015 DTP3 estimates. We identified the African Region countries that had achieved ≥90% DTP3 coverage in 2015, and grouped the remaining countries according to level of DTP3 coverage achieved (80%–89%, 70%–79% and <70%). We used the 2010 and 2015 estimates to calculate the relative change in national DTP3 coverage over the five-year period and compared the relative change in coverage across the Region.

Using the UNPD estimates for surviving infants [9], we calculated weighted average vaccination coverage by income category and Gavi-eligibility status for the years 2000, 2005, 2010 and 2015 and compared the relative change by income category groups and Gavi-eligibility.

We identified African Region countries with particularly good performance using the following three criteria: 1) countries that maintained a national DTP1 and DTP3 coverage ≥90% for the period 2013 to 2015, 2) countries that maintained DTP1-3 dropout ≤10% for the period 2013 to 2015 and 3) countries that maintained a MCV1 coverage ≥90% for the period 2013 to 2015. Countries met all three criteria were defined as positive deviants.

Finally, we reviewed the African Region’s performance regarding the introduction of and coverage with selected vaccines that have become available since 1980 or remain underused. We compared the African Region’s coverage with each of these vaccines to that of the other five WHO Regions and to global coverage.

## Results

### DTP3 and Pol3 coverage trends

**Figure 1:**
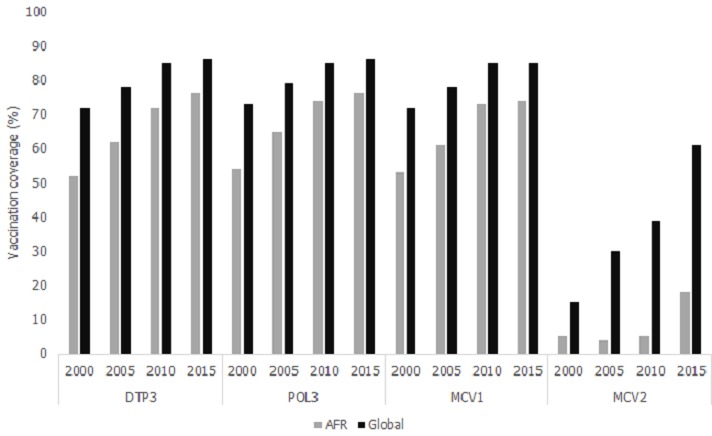
Routine immunization coverage globally and in the African Region, 2000-2015

In 2015, regional DTP3 coverage in the African Region reached 76% from a 2000 level of52%. In comparison, global DTP3 coverage increased from 72% to 86% over the same period (Figure 1). In 2015, national DTP3 coverage for African Region countries ranged from 16% (Equatorial Guinea) to 98% (Rwanda and Tanzania), with 16 (34%) of all 47 African Region countries achieving ≥90% national DTP3 coverage and 6(13%)reaching ≥80% DTP3 coverage in every district. Thirteen of the countries achieving ≥90% national DTP3 coverage had also done so in 2014 and 2013. Of the 31 African Region countries that did not achieve ≥90% national DTP3 coverage in 2015, 16 (34%) had coverage of 80%-89%, 3 (6%) had coverage of 70%-79% and 12 (26%) had coverage of <70%. The 2015 African Region coverage of 76% resulted in 8.1 million children unvaccinated with DTP3 (42% of the global total in 2015); nearly 3.9 million lived in three African Region countries: Nigeria (2.9 million), Democratic Republic of the Congo (0.6 million) and Ethiopia (0.4 million). In 2000, 11.7 million were unvaccinated with DTP3.

From 2010 to 2015, the relative change in national DTP3 coverage varied between -28% and 25% across countries. Eighteen African Region countries experienced a reduction in DTP3 coverage from 2010 to 2015; of these, 4 countries (Equatorial Guinea, Liberia, Angola and Guinea) had a greater than 10% reduction in DTP3 coverage from 2010 to 2015. Over the same period, the African Region Pol3 coverage showed similar patterns as DTP3 coverage (Figure 1). The African Region Pol3 coverage minimally increased, moving from 74% in 2010 to 76% in 2015, with 2015 Pol3 national coverages ranging between 17% (Equatorial Guinea) and 98% (Rwanda and Tanzania).

### MCV1 and MCV2 coverage trends

Estimated MCV1 coverage in the African Region increased from 53% in 2000 to 73% by 2010 and was 74% in 2015 (Figure 1). In comparison, global MCV1 coverage increased from 72% in 2000 to 85% in 2010 and was still 85% in 2015. In 2015, national MCV1 coverage for African Region countries ranged between 20% and 99%, with 12 (26%) countries reaching ≥90% national MCV1 coverage. From 2010 to 2015, 20 African Region countries experienced a decrease in MCV1 coverage, 23 experienced an increase, and 4had no change. Five countries (Swaziland, Kenya, Equatorial Guinea, Eritrea, and Angola) had a greater than 10% decrease in MCV1 coverage from 2010 to 2015. MCV2 coverage in the African Region (including countries that had not yet introduced MCV2) increased from 5% in 2000 to 18% in 2015, with 23 (49%) African Region countries including MCV2 in their 2015 immunization schedule compared to 5 (11%) in 2000. In comparison, 83% of countries globally had introduced MCV2 by 2015, compared to 46% in 2000 and global MCV2 coverage was 61% in 2015, compared to 15% in 2000.

### Analysis by income category and Gavi-eligibility status

When classified by country income category in 2015, 26 African Region countries were low-income, 12 were lower middle-income, 8 were upper middle-income and 1 was high income; these numbers changed from 36, 5, 5, and 0, respectively, in 2000 (Table 1).

**Table 1:** Income category of each country in the World Health Organization African Region by year, 2000, 2005, 2010 and 2015

Country	Income category by year			
	2000	2005	2010	2015
Algeria	LOWER MIDDLE	LOWER MIDDLE	UPPER MIDDLE	UPPER MIDDLE
Angola	LOW	LOWER MIDDLE	LOWER MIDDLE	UPPER MIDDLE
Benin	LOW	LOW	LOW	LOW
Botswana	UPPER MIDDLE	UPPER MIDDLE	UPPER MIDDLE	UPPER MIDDLE
Burkina Faso	LOW	LOW	LOW	LOW
Burundi	LOW	LOW	LOW	LOW
Cabo Verde	LOWER MIDDLE	LOWER MIDDLE	LOWER MIDDLE	LOWER MIDDLE
Cameroon	LOW	LOWER MIDDLE	LOWER MIDDLE	LOWER MIDDLE
Central African Republic	LOW	LOW	LOW	LOW
Chad	LOW	LOW	LOW	LOW
Comoros	LOW	LOW	LOW	LOW
Congo, Democratic Republic of	LOW	LOW	LOW	LOW
Congo, Republic of	LOW	LOWER MIDDLE	LOWER MIDDLE	LOWER MIDDLE
Côte d'Ivoire	LOW	LOW	LOWER MIDDLE	LOWER MIDDLE
Equatorial Guinea	LOWER MIDDLE	UPPER MIDDLE	High	UPPER MIDDLE
Eritrea	LOW	LOW	LOW	LOW
Ethiopia	LOW	LOW	LOW	LOW
Gabon	UPPER MIDDLE	UPPER MIDDLE	UPPER MIDDLE	UPPER MIDDLE
Gambia, The	LOW	LOW	LOW	LOW
Ghana	LOW	LOW	LOWER MIDDLE	LOWER MIDDLE
Guinea	LOW	LOW	LOW	LOW
Guinea-Bissau	LOW	LOW	LOW	LOW
Kenya	LOW	LOW	LOW	LOWER MIDDLE
Lesotho	LOW	LOWER MIDDLE	LOWER MIDDLE	LOWER MIDDLE
Liberia	LOW	LOW	LOW	LOW
Madagascar	LOW	LOW	LOW	LOW
Malawi	LOW	LOW	LOW	LOW
Mali	LOW	LOW	LOW	LOW
Mauritania	LOW	LOW	LOWER MIDDLE	LOWER MIDDLE
Mauritius	UPPER MIDDLE	UPPER MIDDLE	UPPER MIDDLE	UPPER MIDDLE
Mozambique	LOW	LOW	LOW	LOW
Namibia	LOWER MIDDLE	LOWER MIDDLE	UPPER MIDDLE	UPPER MIDDLE
Niger	LOW	LOW	LOW	LOW
Nigeria	LOW	LOW	LOWER MIDDLE	LOWER MIDDLE
Rwanda	LOW	LOW	LOW	LOW
São Tomé and Principe	LOW	LOW	LOWER MIDDLE	LOWER MIDDLE
Senegal	LOW	LOW	LOWER MIDDLE	LOW
Seychelles	UPPER MIDDLE	UPPER MIDDLE	UPPER MIDDLE	HIGH
Sierra Leone	LOW	LOW	LOW	LOW
South Africa	UPPER MIDDLE	UPPER MIDDLE	UPPER MIDDLE	UPPER MIDDLE
South Sudan	..	..	..	LOW
Swaziland	LOWER MIDDLE	LOWER MIDDLE	LOWER MIDDLE	LOWER MIDDLE
Tanzania	LOW	LOW	LOW	LOW
Togo	LOW	LOW	LOW	LOW
Uganda	LOW	LOW	LOW	LOW
Zambia	LOW	LOW	LOWER MIDDLE	LOWER MIDDLE
Zimbabwe	LOW	LOW	LOW	LOW

Shaded cells indicate where country changed income category from previous 5-year time point. Source: World Bank analytical categorizations using country gross national income per capita for given year

**Figure 2:**
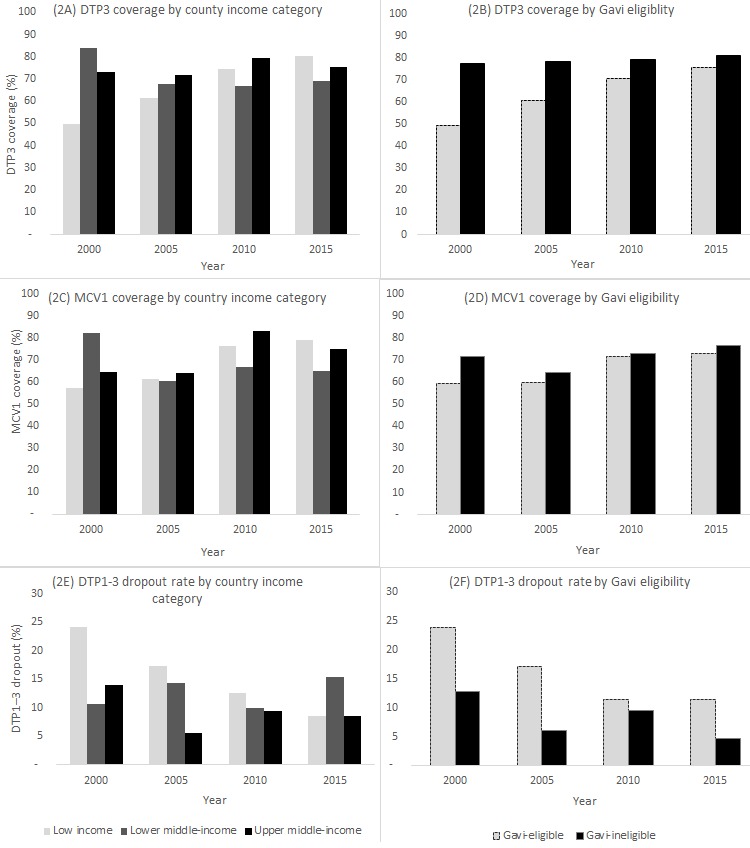
Immunization program performance (MCV1 coverage, DTP3 coverage and DTP1–3 dropout rate) for countries in the African Region 1, by country income category 2 and Gavi-eligibility status 3, 2000–2015

The population-weighted average national DTP3 coverage across low-income African Region countries increased from 50% in 2000 to 80% in 2015 (average annual change, 2.0%), with a lower annual change during 2010-2015, when coverage increased from 74% to 80% (average annual change, 1.2%), than during 2000-2010, when coverage increased from 50% to 74% (average annual change, 2.4%) (Figure 2 A). In lower middle-income countries, the average DTP3 coverage decreased from 84% in 2000 to 69% in 2015 (average annual change,-1.0%), though there was a slight increase from 67% to 69% during 2010-2015. The decrease in lower middle-income countries’ average DTP3 coverage coincided with Nigeria being reclassified as a lower middle-income country instead of a low income country. Nigeria’s 2015 DTP3 coverage of 56% was below the 87% average for the other lower middle-income countries. For lower middle-income countries excluding Nigeria, DTP3 coverage was 84% in 2000, 68% in 2005, 84% in 2010, and 87% in 2015. In comparison, for low income countries excluding Nigeria, DTP3 coverage increased from 55% in 2000 to 69% in 2005, 74% in 2010 and 80% in 2015.

Among upper middle-income countries, a small annual change (average annual change, 0.2%) was observed during 2000-2015 as DTP3 coverage increased from 73% to 76%; however, coverage reached a high of 79% in 2010 before decreasing to 76% by 2015. The average national DTP3 coverage increased from 77% to 81% between 2000 and 2015 (average annual change, 0.3%) for Gavi-ineligible countries and from 49% to 76% (average annual change, 1.8%) for Gavi-eligible countries (Figure 2 B).

African Region MCV1 coverage by income and Gavi-eligibility category exhibited similar trends as DTP3 coverage during the 2000-2015 period (Figure 2 C,D). In low-income African Region countries, MCV1 coverage increased from 57% to 79% (average annual change: 1.6 percentage points) during 2000-2015, while in lower middle-income countries, coverage was 82% in 2000 and 65% in 2015 (average annual change,-1.1%). For lower middle-income countries without Nigeria, MCV1 coverage was 82% in 2000 and 60% in 2005, then reached 81% in 2010 and 80% in 2015 (average annual change, -0.1%). In upper middle-income countries, coverage was 65% in 2000 and reached 75% in 2015 (average annual change,0.7%). For upper middle-income countries, the largest change in MCV1 coverage occurred during 2005-2010 (64% to 83%). Between 2000 and 2015, MCV1 coverage rose from 60% to 73% in Gavi-eligible countries (average annual change, 0.9%) and from 72% to 77% (average annual change, 0.33%) in Gavi-ineligible countries. The MCV1 coverage gap between Gavi-eligible and Gavi-ineligible African Region countries decreased from 12% to 4% during 2000 to 2015.

The average DTP1–3 dropout rate was 24% in 2000 and 9% in 2015 among African Region countries that were low-income in those years, 11% in 2000 and 15% in 2015 in countries that were lower middle-income in those years, and 14% in 2000 and 9% in 2015 among African Region countries that were upper-middle income in those years (Figure 2 E). The average DTP1–3 dropout rate decreased from 13% to 5% from 2000 to 2015 for Gavi-ineligible countries and from 24% to 11% for Gavi-eligible countries (Figure 2 F).

### Positive deviance analysis

During 2013-2015, eleven African Region countries met all three positive deviance analysis thresholds: national DTP1 and DTP3 coverage ≥90%, DTP1–3 dropout <10% and MCV1 coverage ≥90% for each of the last three years. Of these, four were low-income countries in 2015 (Rwanda, Burundi, The Gambia and the United Republic of Tanzania), three were lower middle-income (Lesotho, Sao Tome and Principe and Cabo Verde), three were upper middle-income countries (Algeria, Botswana, and Mauritius) and one was a high-income country (Seychelles). Of these 11 countries, The Gambia, Mauritius, Rwanda and Sao Tome and Principe also achieved ≥80% DTP3 coverage in 100% of districts.

Two additional countries in the African Region achieved national DTP1 and DTP3 coverage ≥90% and DTP1–3 dropout <10% for the last three years, but did not reach the positive deviance threshold of MCV1 coverage ≥90% (Eritrea and Swaziland).

### Vaccine introductions

Several vaccines have been added to the WHO recommended schedule since the inception of the Expanded Programme on Immunization (EPI) in 1974. By 2015, 23 (49%) African Region countries had introduced MCV2, while 37 (79%) had introduced PCV and 29 (62%) had introduced rotavirus vaccine. Regional coverage for Hib3, PCV3, RCV1 and Rota-last increased in the African Region between 2010 and 2015 (Table 2), as 33 African Region countries introduced PCV, 27 introduced rotavirus vaccine, 7 introduced Hib, and 6 introduced RCV1. African Region PCV3 reached 59% in 2015 compared to global coverage of 37%. Among the six WHO Regions, the African Region ranked fourth in 2015 for Hib3 coverage and second for both PCV3 and Rota-last coverage (data not shown). However, African Region RCV1 coverage was the lowest among all regions at 12%. In the African Region, the number and proportion of countries reaching 90% national coverage in 2015 with Hib3, PCV3, Rota-last and RCV1 vaccines were 16 (34%), 6 (13%), 6 (13%) and 5 (11%), respectively. Of the four positive deviant, low-income countries (as of 2015), all achieved ≥90% coverage with Hib3, PCV3, and Rota-last in 2015; Tanzania and Rwanda also achieved ≥90% coverage with RCV1 in 2015, while Burundi and The Gambia had yet to introduce RCV1.

**Table 2:** African region and global coverage of recently introduced vaccines, 2010 and 2015

	**African Region coverage (%)**			**Global coverage (%)**		
Vaccine	2010	2015	Change	2010	2015	Change
Hib3	61	76	+15	42	64	+22
PCV3	4	59	+55	11	37	+26
RCV1	0	12	+12	35	46	+11
Rota	2	41	+39	8	23	+15

Definitions: Rota=final dose of rotavirus vaccine; Hib3=third dose of *Haemophilus influenzae* type b vaccine; PCV3=third dose of pneumococcal conjugate vaccine; RCV1=first dose of rubella-containing vaccine

## Discussion

In this report, we provide an update on the African Region’s progress and challenges in reaching GVAP vaccination coverage goals intended to help extend the benefits of immunization equitably to all people. Over the past 15 years, African Region vaccination coverage increases have outpaced global coverage increases, with a 24% increase in DTP3 coverage and a 21% increase in MCV1 coverage, while the number of children unvaccinated with DTP3 in the Region decreased to a low of 8.1 million in 2015 from 11.8 million in 2000.However, since 2010 MCV1 and DTP3 vaccination coverage has improved little in the African region, with lower middle income countries having the lowest DTP3 coverage among country income categories in 2015, largely due to Nigeria’s DTP3 coverage. Additionally, within-country geographic inequities are substantial, with only 13% of countries achieving ≥80% DTP3 coverage in every district in 2015. Despite the large increases in DTP3 and MCV1 regional coverage during 2000-2015, the annual rate of change has slowed substantially during 2010-2015, indicating a great risk of not reaching GVAP goals if the pattern continues.

In this African Region analysis, we identified inequities in vaccination coverage among country income groups, between Gavi-eligibility groups, and at the national and subnational levels. In particular, we found that lower middle-income African Region countries had lower DTP3 coverage than low-income or upper middle-income African Region countries. The lower middle-income countries also had lower MCV1 coverage and higher DTP1-3 dropout rates than low-income or upper middle-income African Region countries in 2015. We also found that despite considerable progress in narrowing the gap between Gavi-eligible and Gavi-ineligible countries across program performance indicators, a gap still exists. The continued existence of this gap indicates the need for sustained, long-term investment in strengthening immunization systems. Ensuring adequate funding for national immunization programs across the African Region is particularly critical at this time as funds from the Global Polio Eradication Initiative are beginning to subside and increasing numbers of middle income countries are likely to reach the transition phase out of Gavi support in the coming years

Despite overall substantial improvement in coverage since 2000, we noted concerning temporal trends as 43% and 38% of African Region countries had a decrease in MCV1 and DTP3 coverage, respectively, during 2010-2015. The slowed rate of increase in DTP3 coverage among African Region countries as a whole during 2010-2015 (0.8% average annual change) compared to 2000-2010 (2% average annual change) is similar to patterns seen in other regions [15]. This pattern suggests incremental improvements in coverage may be easier at low levels of coverage than high ones [15], particularly because as coverage rises continued improvements may depend on changes in national infrastructure (e.g., roads, telecommunications), health sector infrastructure (e.g., facilities, supply chain), or health staff (e.g., education) that require considerable time and resources. In addition, these observations suggest that considerable patience and persistence may be required to reach high coverage targets. Understanding the amount of additional funding required to achieve progress at higher baseline levels of coverage and how immunization programs can most efficiently utilize funds and other resources to improve their performance is an important operational research area.

Our findings about the geographic inequities in immunization coverage within countries highlight one of the many types of inequity within countries that lead to less than optimal immunization coverage at the country level [16]. Analyses of data from other sources such as coverage surveys and Demographic and Health Surveys have revealed a wide range of inequities at the subnational level similar to the disparity of WHO/UNICEF coverage estimates at the national and Regional levels. Previous studies have shown coverage to be as much as 60% higher among urban children than rural children [17]. About one in three countries have substantial coverage disparities associated with income level, with at least a 20% gap for DTP and MCV coverage between the richest and the poorest quintiles [18]. The disparities in coverage vary between and within countries and WHO Regions [17],and inequities persist in a large majority of Gavi-eligible countries [19]. Over the last 10 years, there has been considerable progress in improving equity in immunization coverage as the lowest wealth quintile and the least educated have seen the largest improvements in coverage. However, the lower middle and middle quintiles have benefited less from these improvements and warrant greater attention in the future [18].

Using our criteria for positive deviance, we identified four countries that were low-income in 2015 and three countries that were lower middle-income countries in 2015 that were outperforming their peers in reaching and sustaining high vaccination coverage. In 2015, of the positive deviant countries, Rwanda was the only low-income country to achieve ≥80% DTP3 coverage in every district as well as coverage ≥90% for the more recently introduced vaccines included in our analysis (Hib, PCV3, RCV1, and Rota). Nearly reaching this benchmark, Tanzania achieved ≥90% coverage for all the latter vaccines, and ≥80% DTP3 coverage in 92% of its districts. The success in Rwanda has been attributed to several key factors including country ownership and high-level commitment, engagement with village-based community health workers, recognition of immunization as a gateway to access other integrated primary healthcare services, and the creation of a short message service (SMS) text message system for registering pregnancies and births [20, 21]. Rwanda’s commitment to immunization is reflected in its early introduction of new vaccines, which has also given the country more time to increase coverage with those vaccines compared to countries that have introduced them recently.

To achieve immunization and equity goals, the needs and reasons for success of low- and middle-income WHO Regions, countries, districts, and individuals will have to be better understood and addressed [22]. The experience from low-income countries that are making progress in line with GVAP goals, such as Rwanda, should be widely shared and applied. Further context-specific quantitative and qualitative research and tools are required to map gaps in coverage and immunity over time, explore why inequities persist, and determine how best to address them. In the African Region, supporting the evolution of the Reach Every District (RED) strategy into a Reach Every Child (REC) strategy can help address subnational inequities in coverage [23]. A REC strategy can be facilitated by strong microplanning that incorporates fixed site and outreach sessions targeting underserved populations, such as mobile and border groups, non-citizens and undocumented migrants. Improving the implementation of birth registration may also prove beneficial to microplanning efforts, since less than half of the children in the African Region are registered at birth [24, 25]. Clear communications delivered by multiple messengers to stimulate community demand for immunization in all population groups are critical to ensuring that caregivers understand the value of vaccination as well as the risks and see it as a human right [24] and a community responsibility. Establishing strengthened equity-oriented health information systems, including the improved use of mobile technologies, with high-quality immunization data to the individual levelis an important prerequisite to ensuring that policy and practice can be appropriately formulated to achieve immunization coverage targets. For example, where the rate of improvement in coverage slows, prompt efforts are needed to address subnational pockets of low coverage.

This analysis has limitations. While WHO/UNICEF estimates of vaccination coverage are generally thought to best reflect true vaccination coverage, administrative sources have been shown both to overestimate and to underestimate coverage compared to coverage surveys [26]. Although administrative coverage estimates can be more timely and convenient, inaccuracies in either the numerator (number of doses administered) or denominator (census data and projections) can render them unreliable. Several examinations of equity have been performed using coverage survey data, which has the advantage that it is not reliant on the accuracy of administrative numerator, denominator or target population estimates. However, coverage surveys are costly and cannot provide the timely information required to guide immunization programs [27]. As the number of recommended vaccines increases and the use of home vaccination cards declines in some countries, the risk of bias in parental recall may increase [28]. Furthermore, because of the inherent imprecise nature of the coverage estimates, the data in this analysis were examined for every fifth year. An alternative approach would be to use the annual coverage estimates with a data smoothing technique.

## Conclusion

The described inequities in vaccination coverage by region, country and at the subnational level are an important barrier to achieving higher coverage and disease control and elimination goals. Moving forward, successful immunization programs will need to ensure high-level political commitment to immunization, ownership and stewardship of the program, and the financial and human resource capacity to institute a wide range of innovative and cohesive strategies to reach every child and to stimulate demand for vaccination services in all communities. The February 2016 Addis Declaration on Immunization in which African heads of state committed to increasing domestic resources for immunization and improving the effectiveness and efficiency of immunization programs is a sign that the necessary political commitment exists [29]. The fulfillment of the pledges in the declaration will be essential to maximizing the benefits from immunization in the African Region.

### What is known about this topic

- Childhood immunization is a safe, effective and cost-effective intervention that is critical to reducing infant and under-five morbidity and mortality. It is also a key part of the struggle against antimicrobial resistance and can be used as a tool in health system strengthening;
- Equity is a key component of the strategies outlined by the Global Vaccine Action Plan 2011-2020 and the UN 2030 Agenda for Sustainable Development;
- The Global Vaccine Action Plan 2011-2020 called for all countries to reach and sustain 90% national coverage and 80% coverage in all districts for the third dose of diphtheria, tetanus, and pertussis vaccine (DTP3) by 2015 and 90% national coverage and 80% coverage in all districts for all vaccines in national immunization schedules by 2020.

### What this study adds

- As yet, no countries in the African Region are meeting the 2020 coverage targets;
- During 2013-2015, eleven African Region countries achieved national DTP1 and DTP3 coverage ≥90%, DTP1–3 dropout ≤ 10% and MCV1 coverage ≥ 90% for each of the three years; of these 11 countries, only Rwanda, a low income country, achieved 80% DTP3 coverage in all districts and ≥90% coverage for Hib, PCV3, RCV and Rota in 2015;
- Despite improvements in immunization coverage in the African Region since 2000, more progress is needed in lower middle-income and upper middle-income countries as well as in low income countries.

## Competing interests

The authors declare no competing interest.
